# Supplementary figures and images for: Adrenal SGLT1 or SGLT2 as predictors of atherosclerosis under chronic stress based on a computer algorithm
Source: PeerJ. 2023 Aug 30;11:e15647. doi: 10.7717/peerj.15647 (PMC10474830; doi:10.7717/peerj.15647)

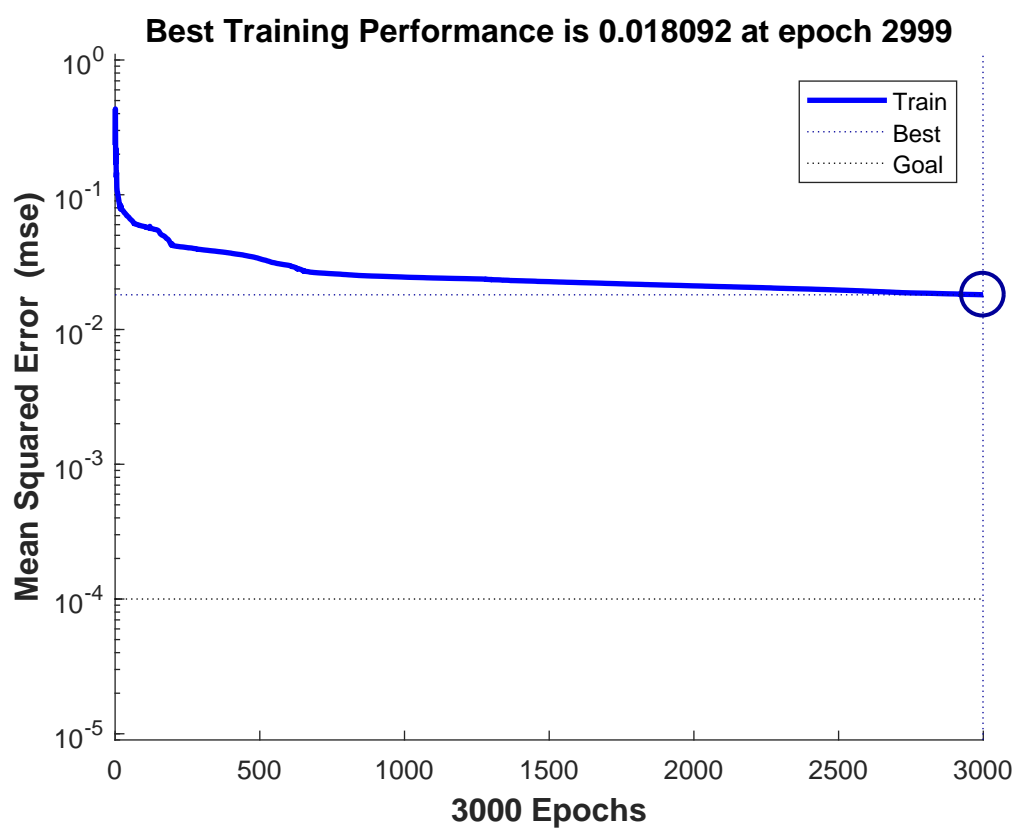

Supplement: Supplemental Information 1 [file peerj-11-15647-s001.zip › Raw Data/Back propagation (BP) neural network raw data/1.Edema/1.edema P value.pdf]

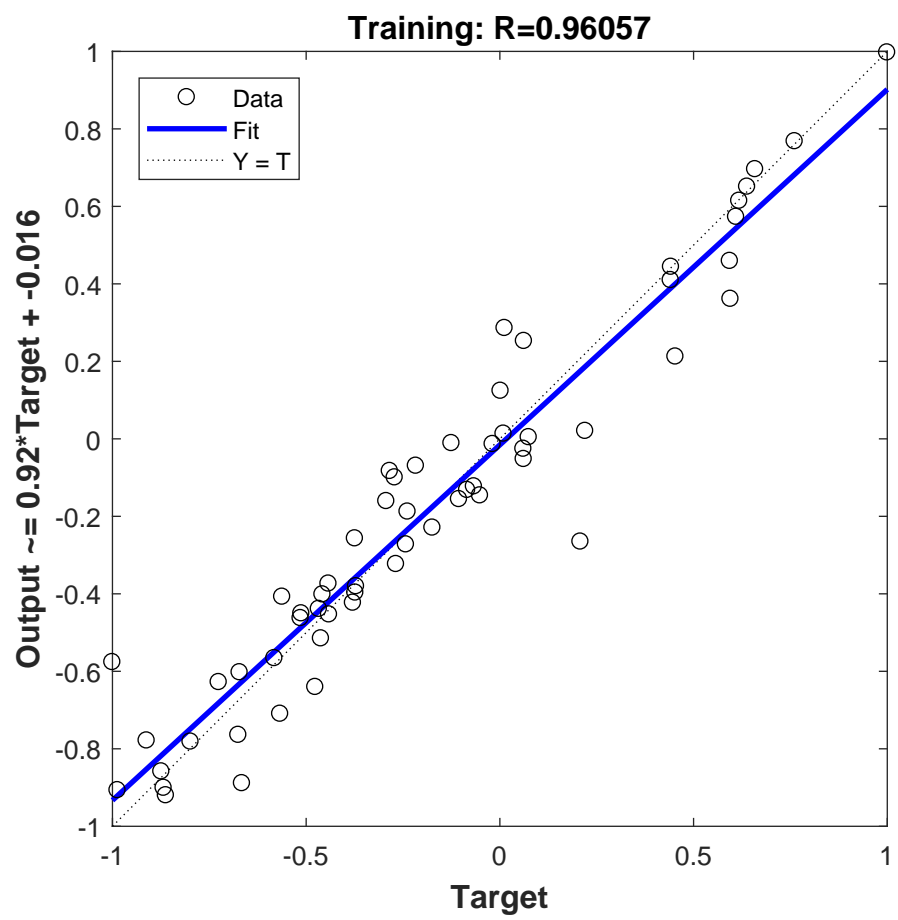

Supplement: Supplemental Information 1 [file peerj-11-15647-s001.zip › Raw Data/Back propagation (BP) neural network raw data/1.Edema/2.edema regression.pdf]

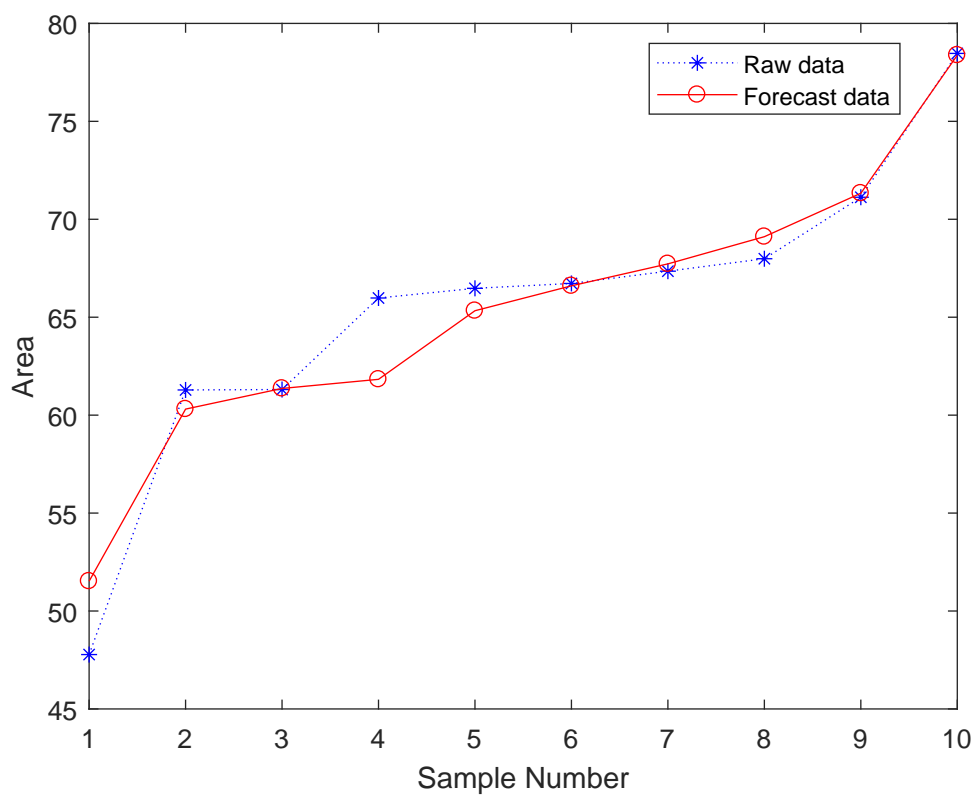

Supplement: Supplemental Information 1 [file peerj-11-15647-s001.zip › Raw Data/Back propagation (BP) neural network raw data/1.Edema/3.edema error comparison.pdf]

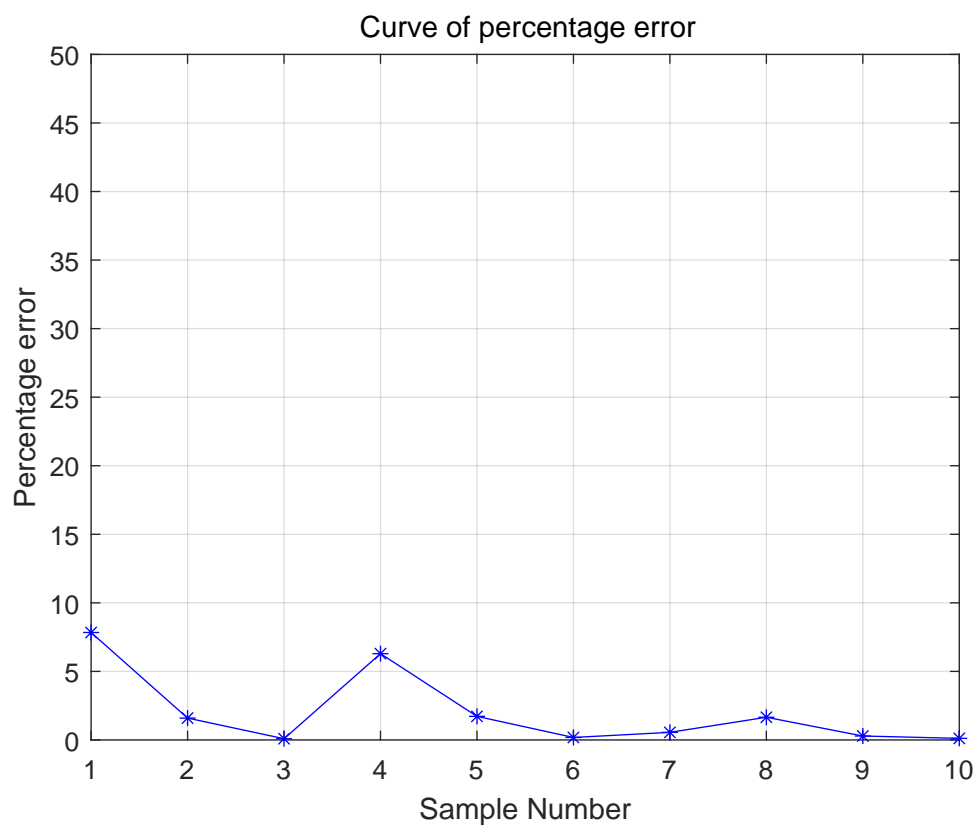

Supplement: Supplemental Information 1 [file peerj-11-15647-s001.zip › Raw Data/Back propagation (BP) neural network raw data/1.Edema/4.edema error contrast percentage.pdf]

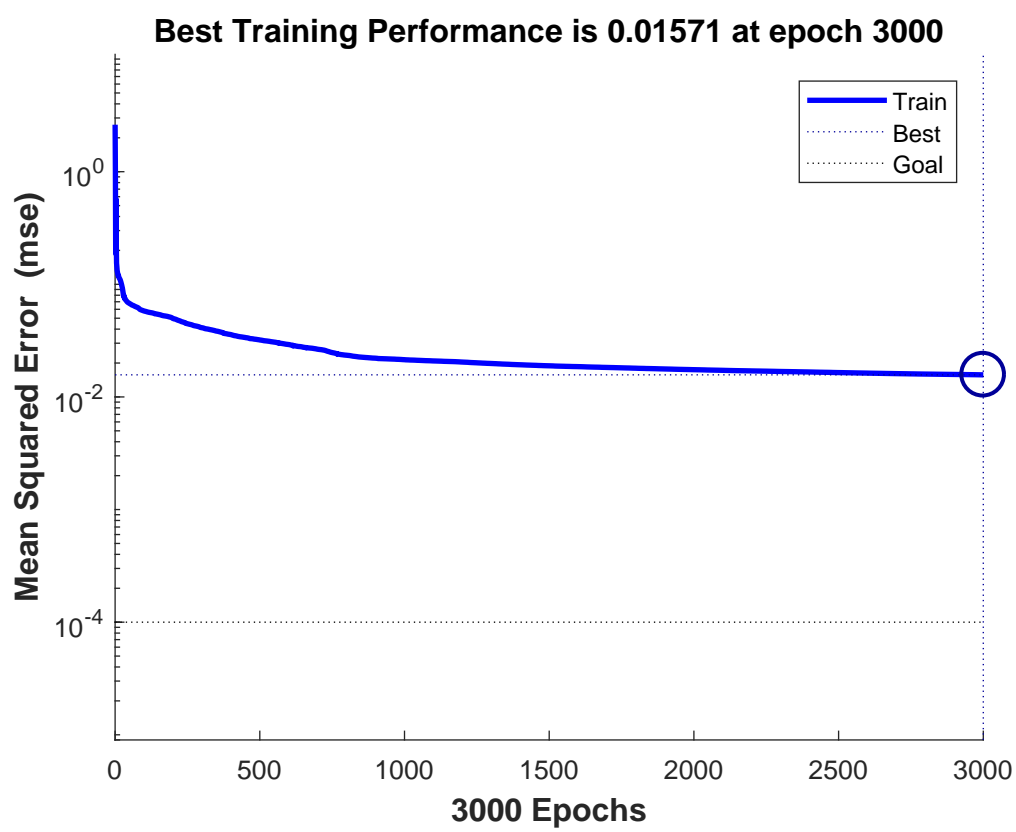

Supplement: Supplemental Information 1 [file peerj-11-15647-s001.zip › Raw Data/Back propagation (BP) neural network raw data/2.Reticulated fibers/1.Reticular fiber P value.pdf]

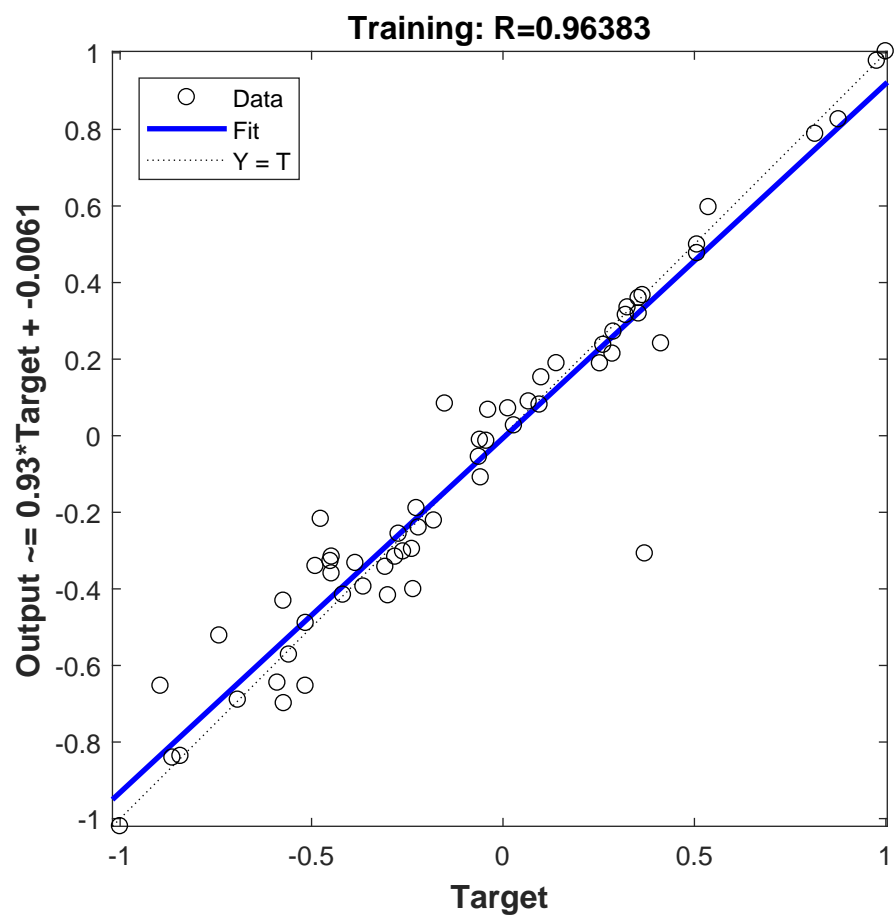

Supplement: Supplemental Information 1 [file peerj-11-15647-s001.zip › Raw Data/Back propagation (BP) neural network raw data/2.Reticulated fibers/2. Reticular fiber regression.pdf]

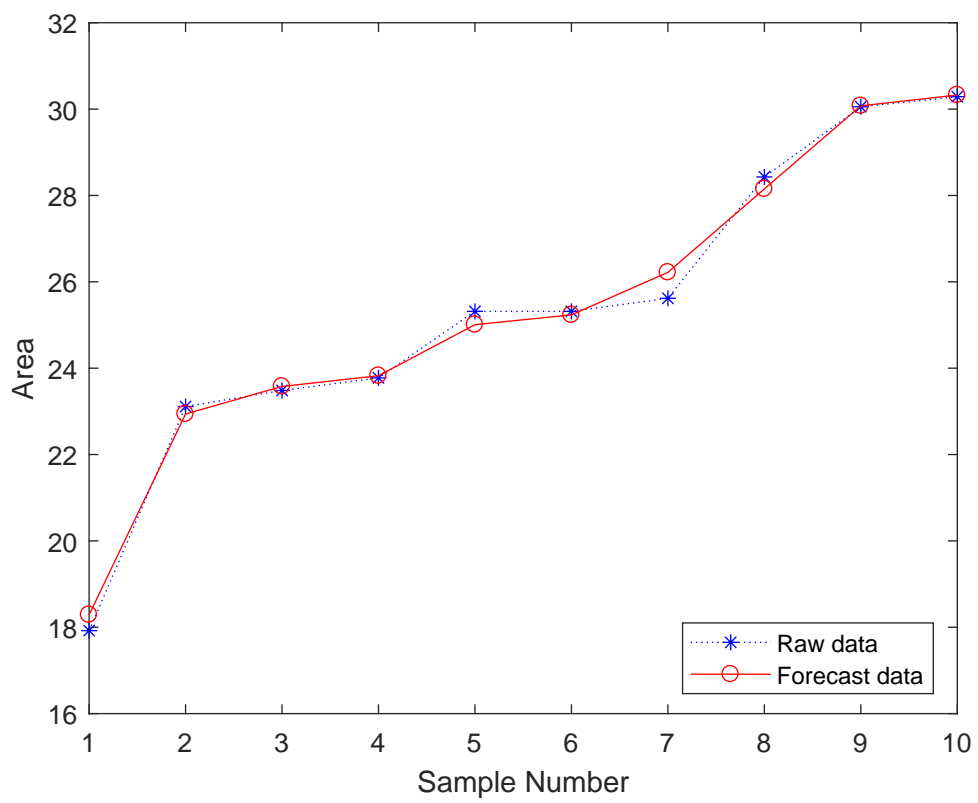

Supplement: Supplemental Information 1 [file peerj-11-15647-s001.zip › Raw Data/Back propagation (BP) neural network raw data/2.Reticulated fibers/3.Error comparison of reticulated fiber.pdf]

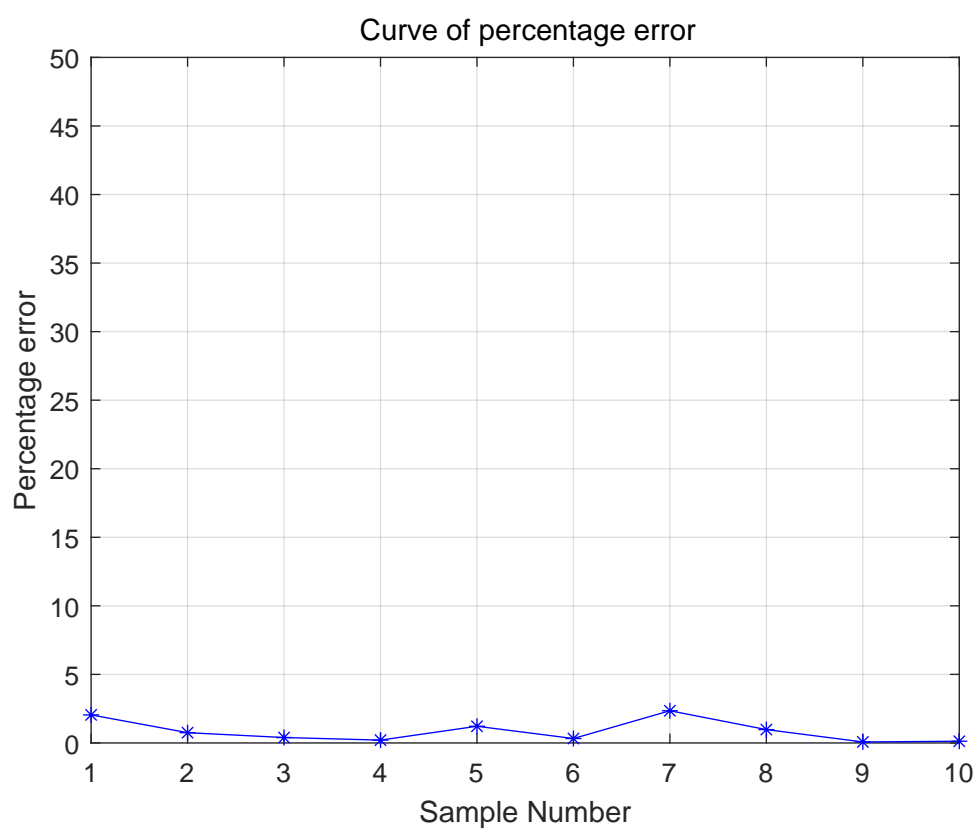

Supplement: Supplemental Information 1 [file peerj-11-15647-s001.zip › Raw Data/Back propagation (BP) neural network raw data/2.Reticulated fibers/4.Error of reticulated fiber.pdf]

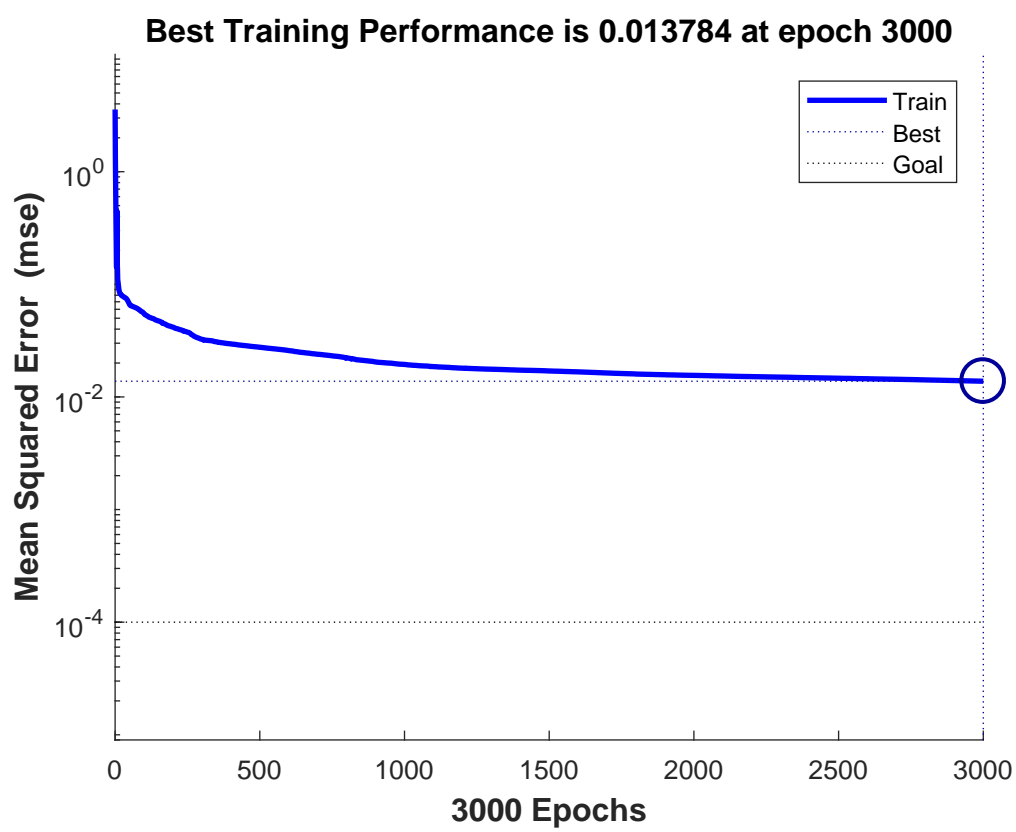

Supplement: Supplemental Information 1 [file peerj-11-15647-s001.zip › Raw Data/Back propagation (BP) neural network raw data/3.Glycogen content/1.Glycogen content P value.pdf]

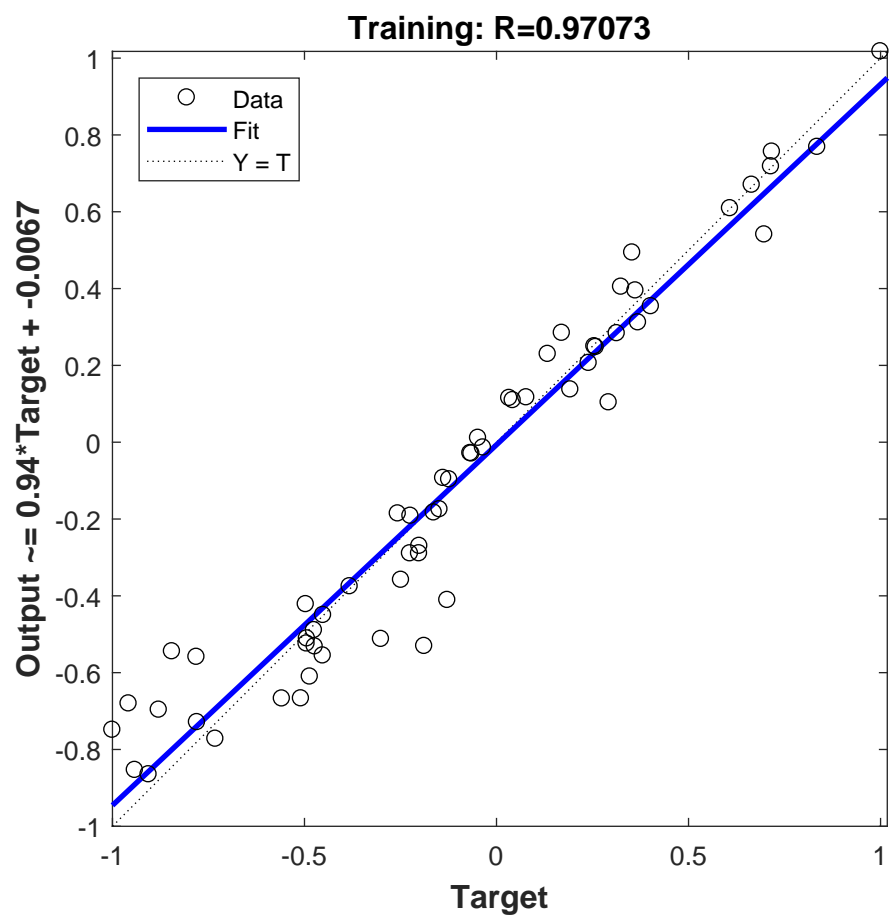

Supplement: Supplemental Information 1 [file peerj-11-15647-s001.zip › Raw Data/Back propagation (BP) neural network raw data/3.Glycogen content/2.Glycogen content regression.pdf]

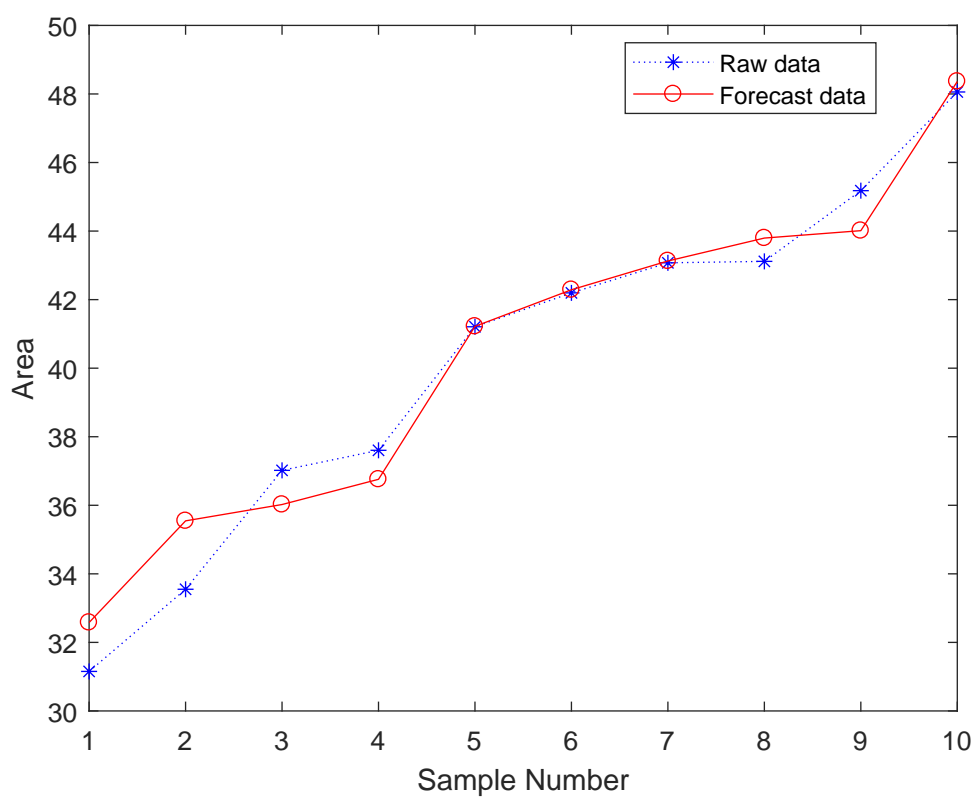

Supplement: Supplemental Information 1 [file peerj-11-15647-s001.zip › Raw Data/Back propagation (BP) neural network raw data/3.Glycogen content/3.Percentage error of glycogen content.pdf]

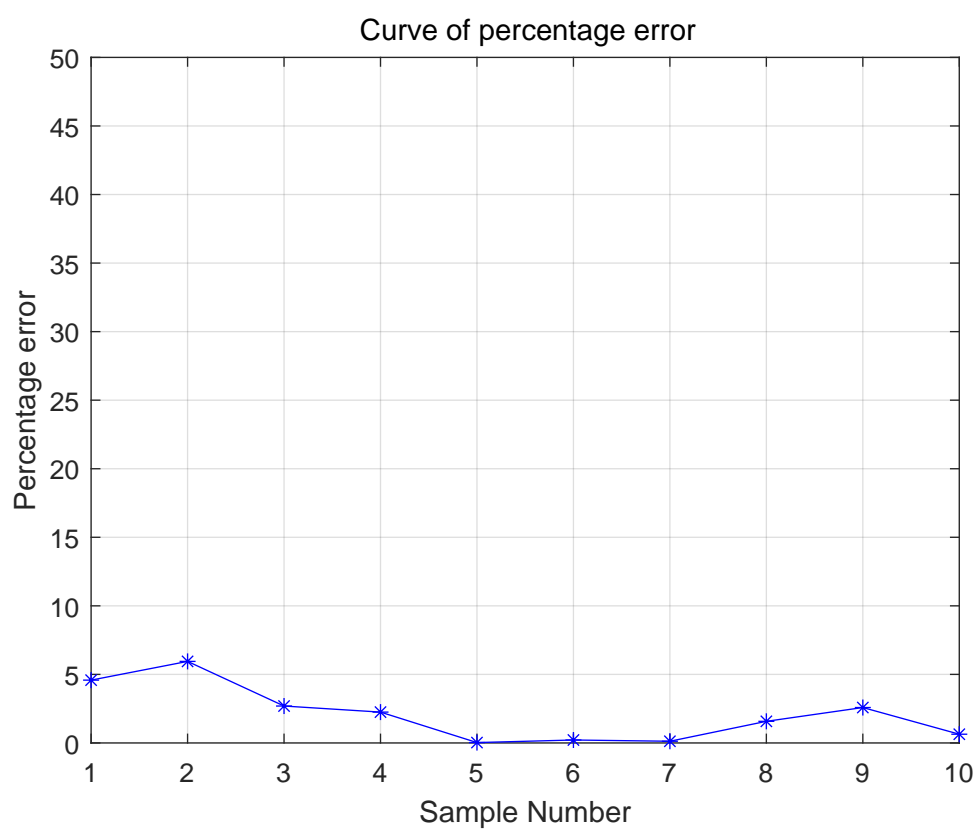

Supplement: Supplemental Information 1 [file peerj-11-15647-s001.zip › Raw Data/Back propagation (BP) neural network raw data/3.Glycogen content/4.Error of glycogen content.pdf]

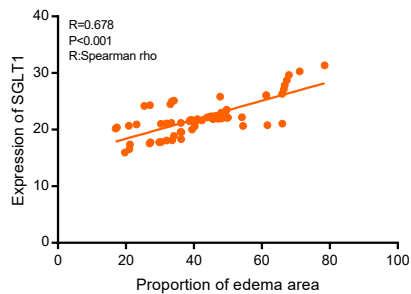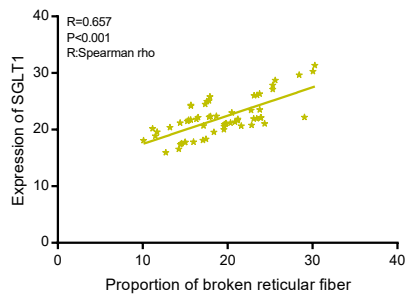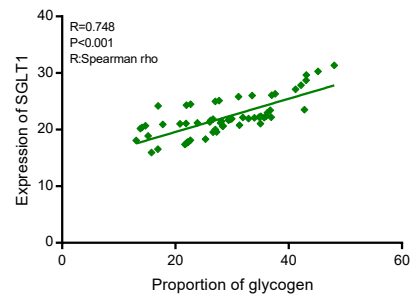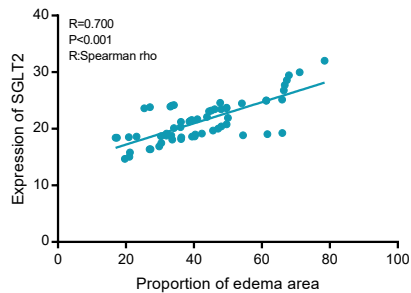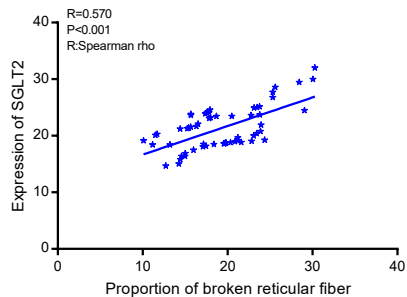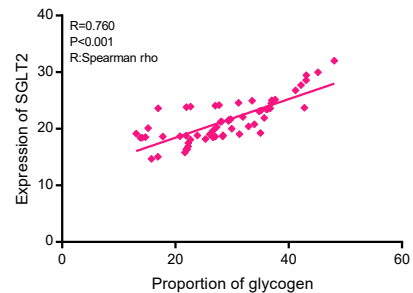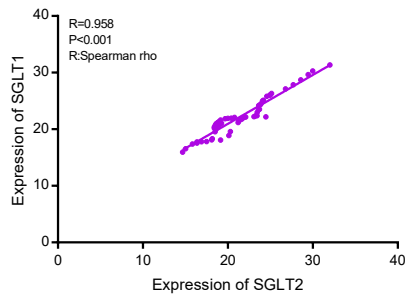

Supplement: Supplemental Information 1 [file peerj-11-15647-s001.zip › Raw Data/Edema, reticulocyte fiber breakage, glycogen content in relation to SGLT1 and SGLT2 expression/Edema, reticulocyte fiber breakage, glycogen content in relation to SGLT1 and SGLT2 expression.pdf]

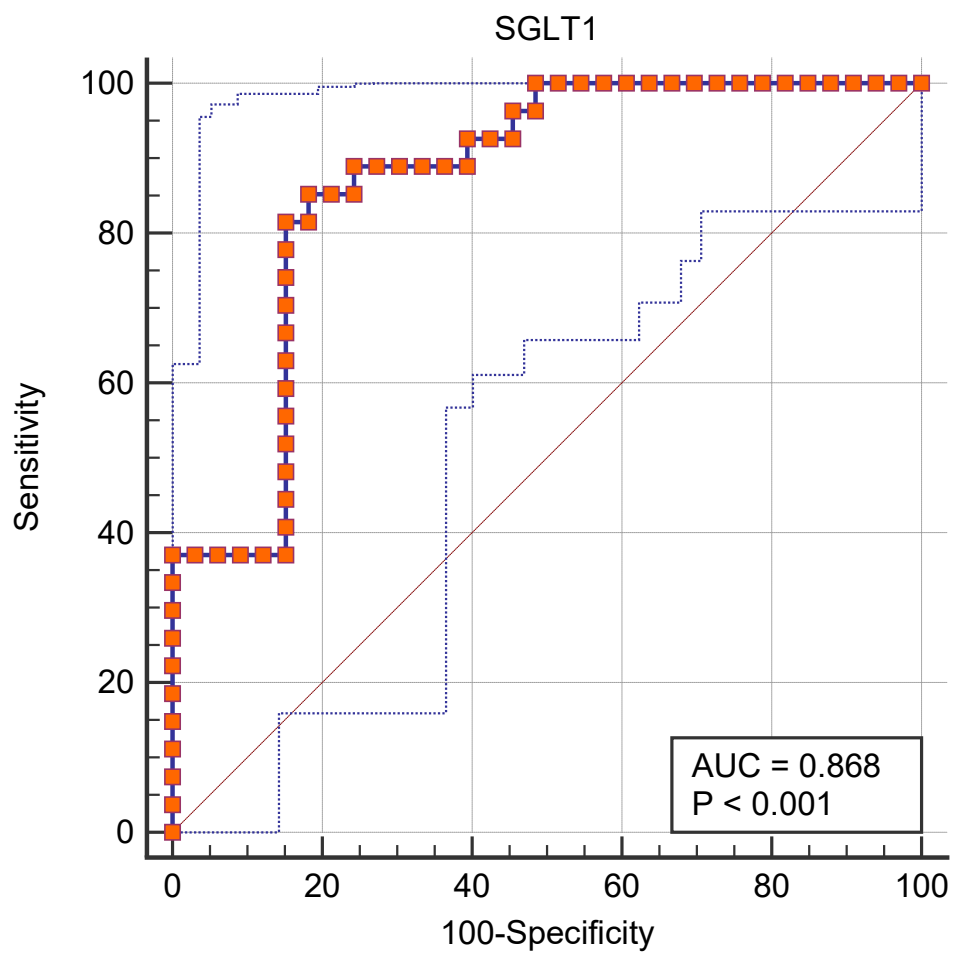

Supplement: Supplemental Information 1 [file peerj-11-15647-s001.zip › Raw Data/ROC raw data/1.SGLT1-edema.pdf]

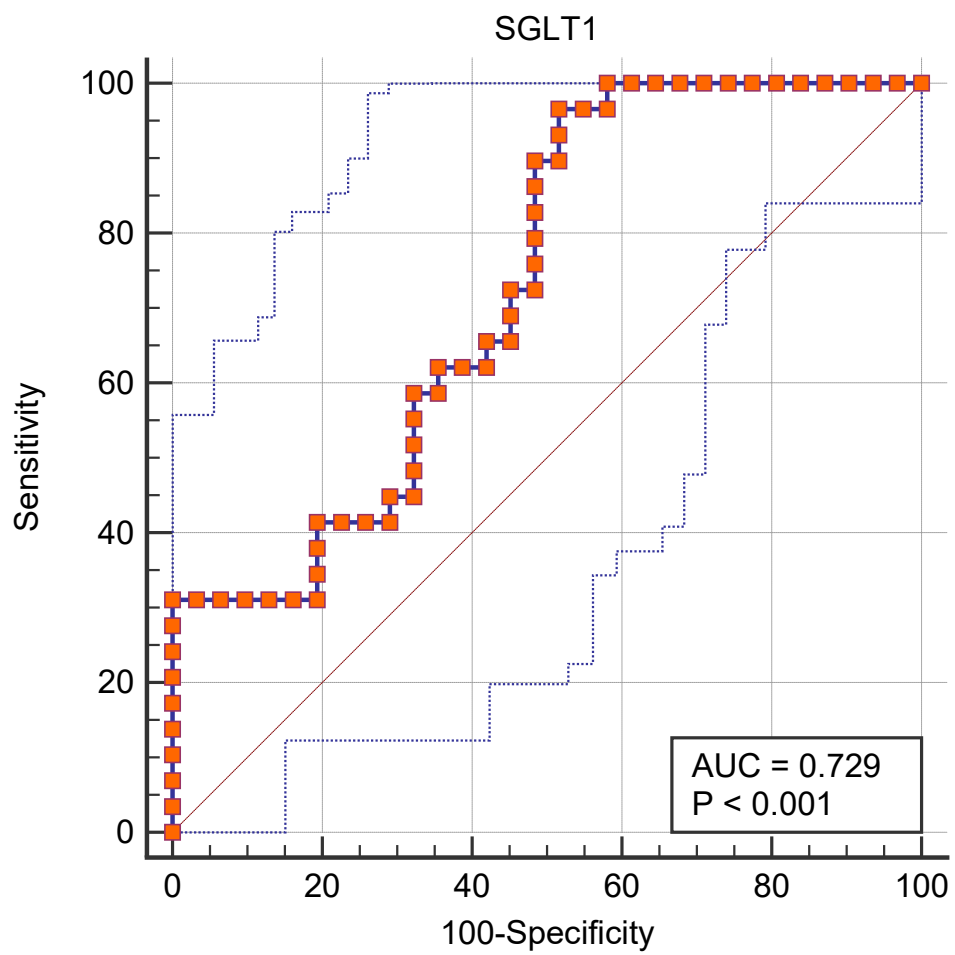

Supplement: Supplemental Information 1 [file peerj-11-15647-s001.zip › Raw Data/ROC raw data/2.SGLT1-reticular fiber .pdf]

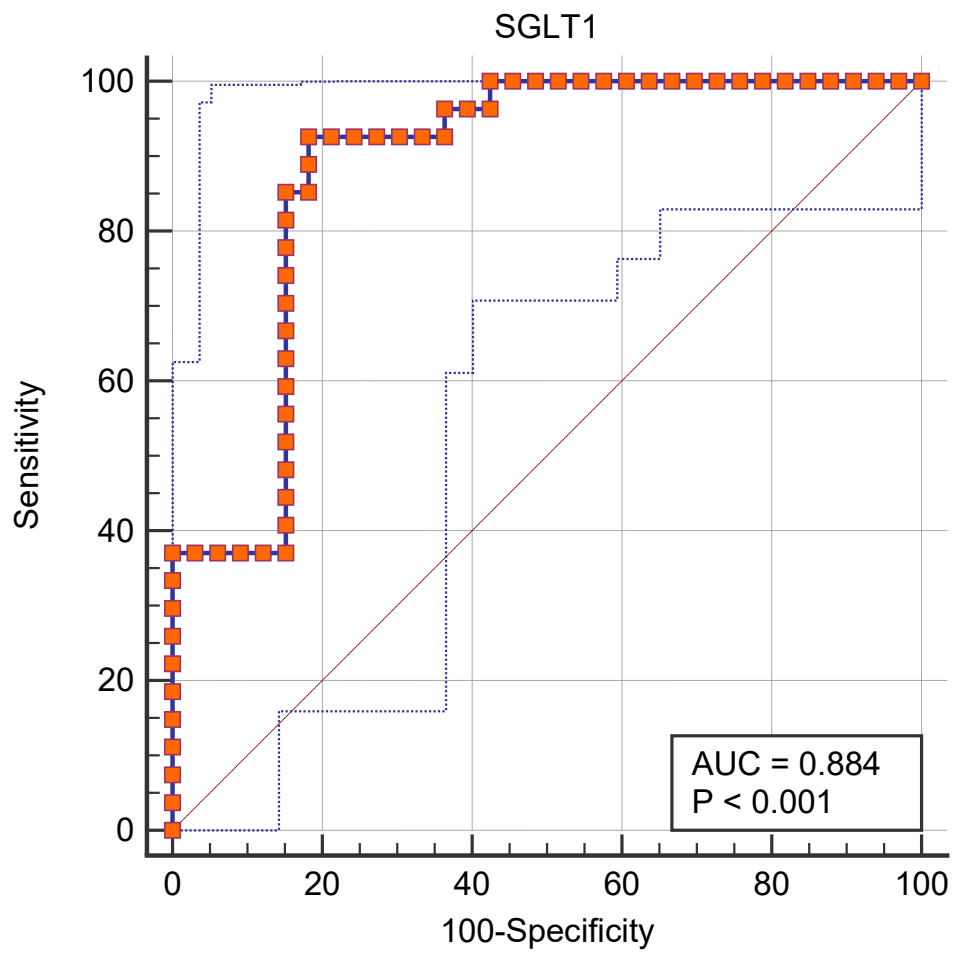

Supplement: Supplemental Information 1 [file peerj-11-15647-s001.zip › Raw Data/ROC raw data/3.SGLT1-glycogen content.pdf]

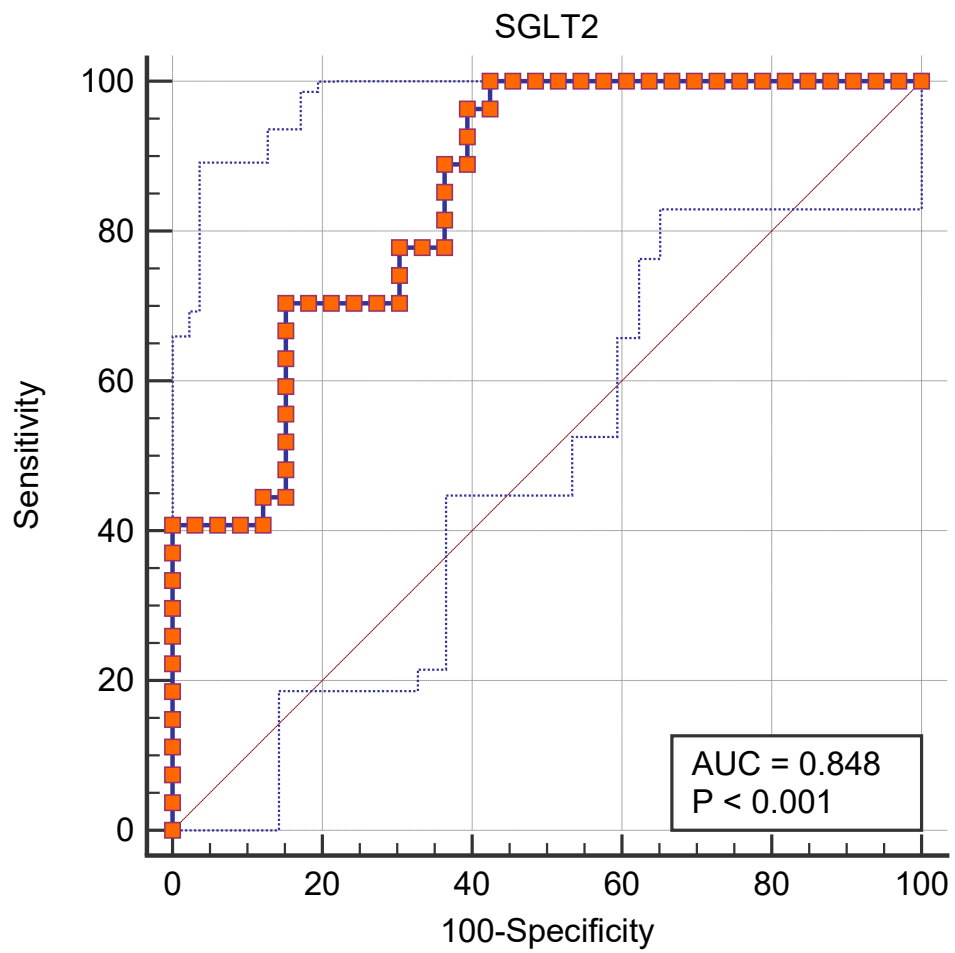

Supplement: Supplemental Information 1 [file peerj-11-15647-s001.zip › Raw Data/ROC raw data/4.SGLT2-edema.pdf]

# SGLT2

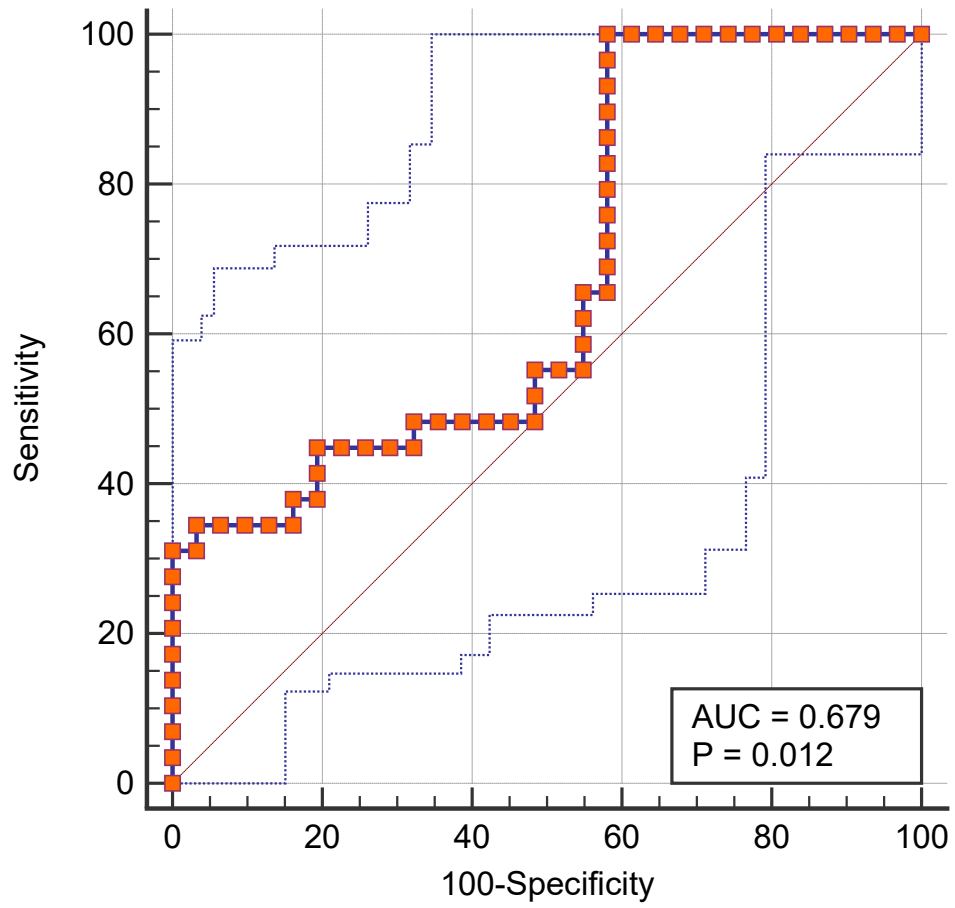

Supplement: Supplemental Information 1 [file peerj-11-15647-s001.zip › Raw Data/ROC raw data/5.SGLT2-reticular fiber .pdf]

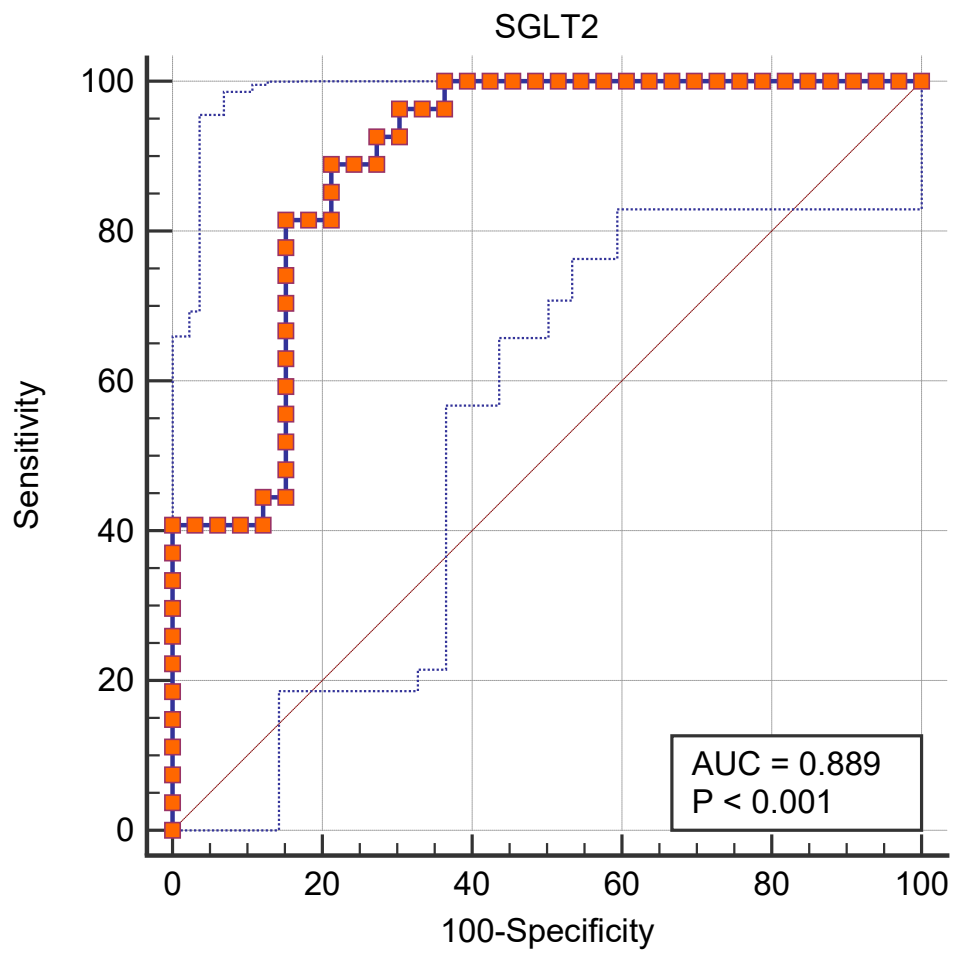

Supplement: Supplemental Information 1 [file peerj-11-15647-s001.zip › Raw Data/ROC raw data/6.SGLT2-glycogen content.pdf]

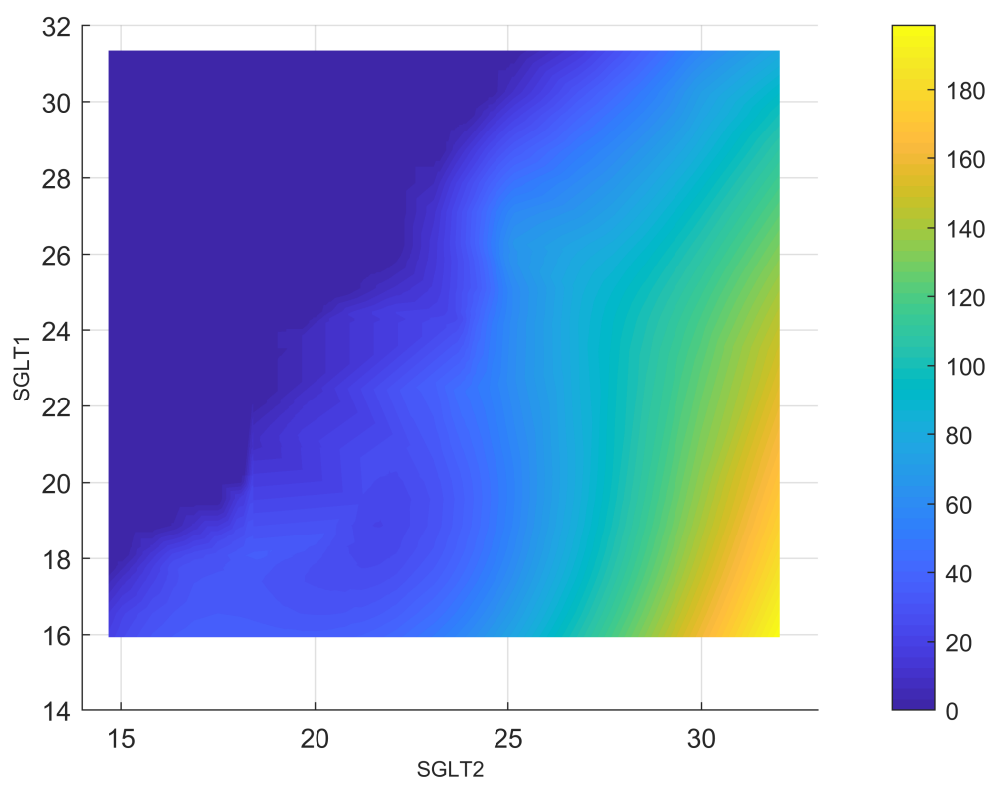

Supplement: Supplemental Information 1 [file peerj-11-15647-s001.zip › Raw Data/Raw data related to interpolation algorithm/1.edema/A high-risk warning indicator for adrenal edema.pdf]

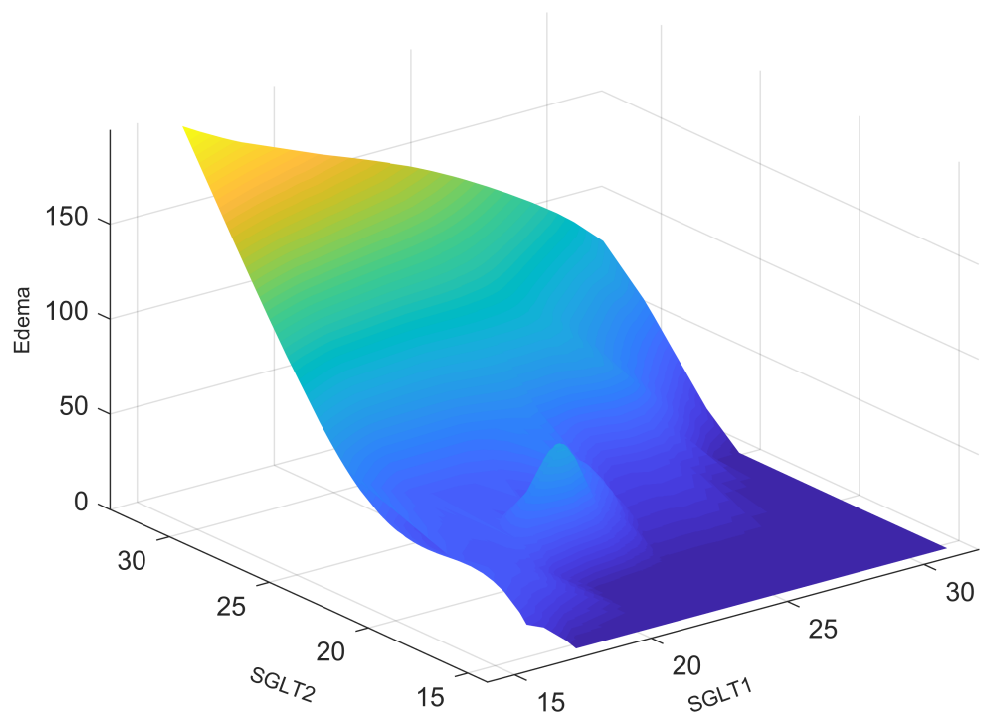

Supplement: Supplemental Information 1 [file peerj-11-15647-s001.zip › Raw Data/Raw data related to interpolation algorithm/1.edema/three-dimensional (3D) stereogram.pdf]

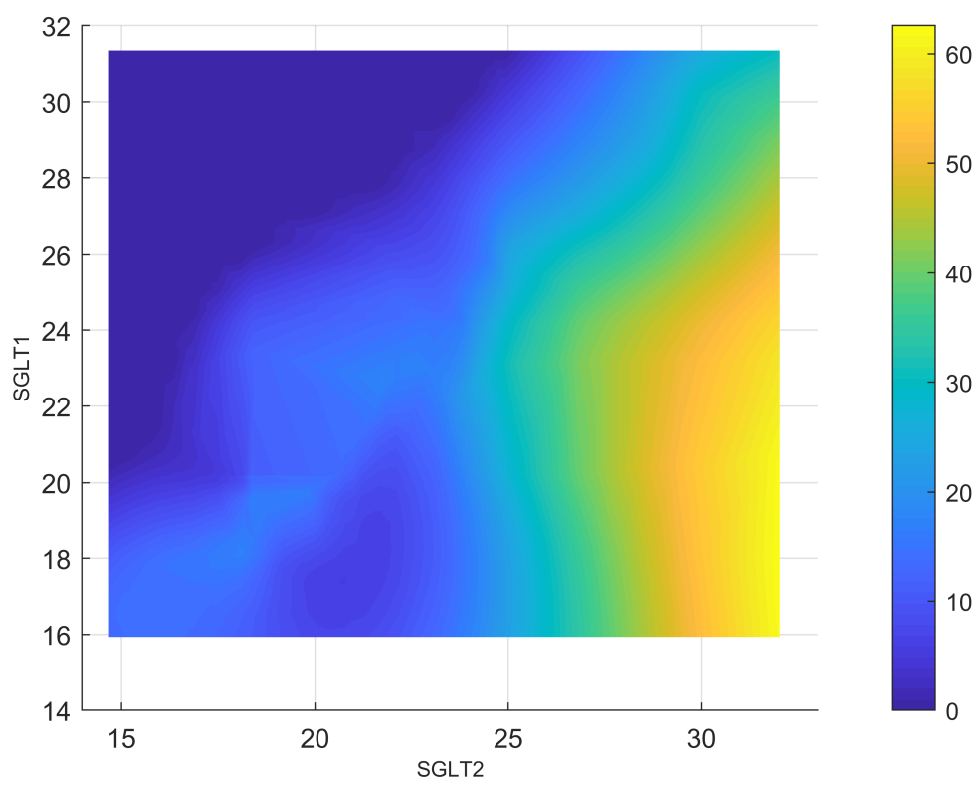

Supplement: Supplemental Information 1 [file peerj-11-15647-s001.zip › Raw Data/Raw data related to interpolation algorithm/2.Reticulated fibers/A high-risk warning indicator for reticulated fibers.pdf]

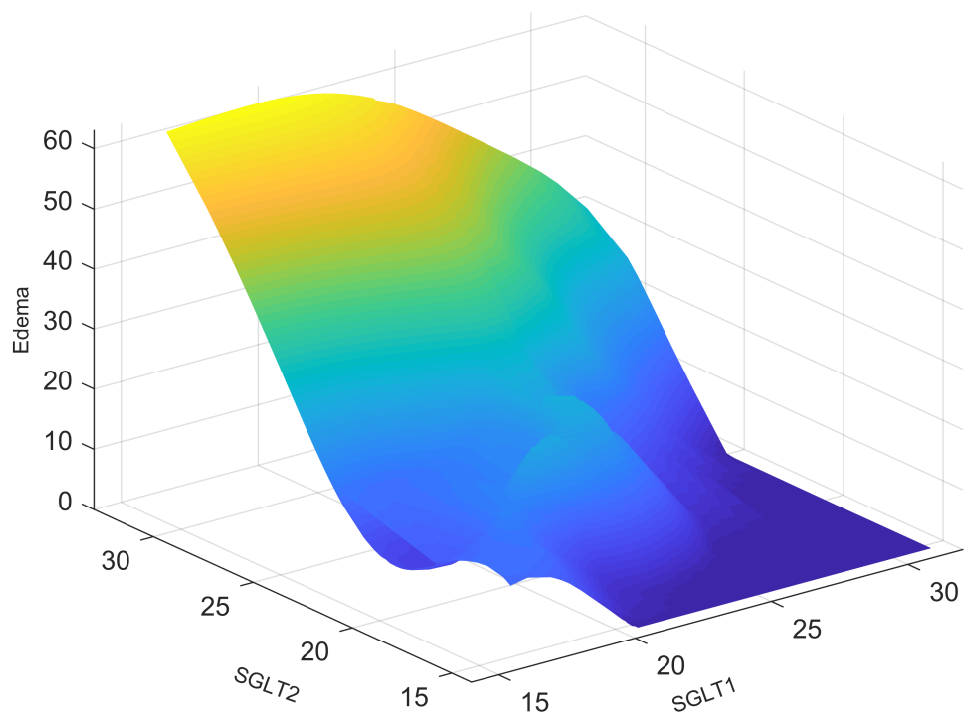

Supplement: Supplemental Information 1 [file peerj-11-15647-s001.zip › Raw Data/Raw data related to interpolation algorithm/2.Reticulated fibers/three-dimensional (3D) stereogram.pdf]

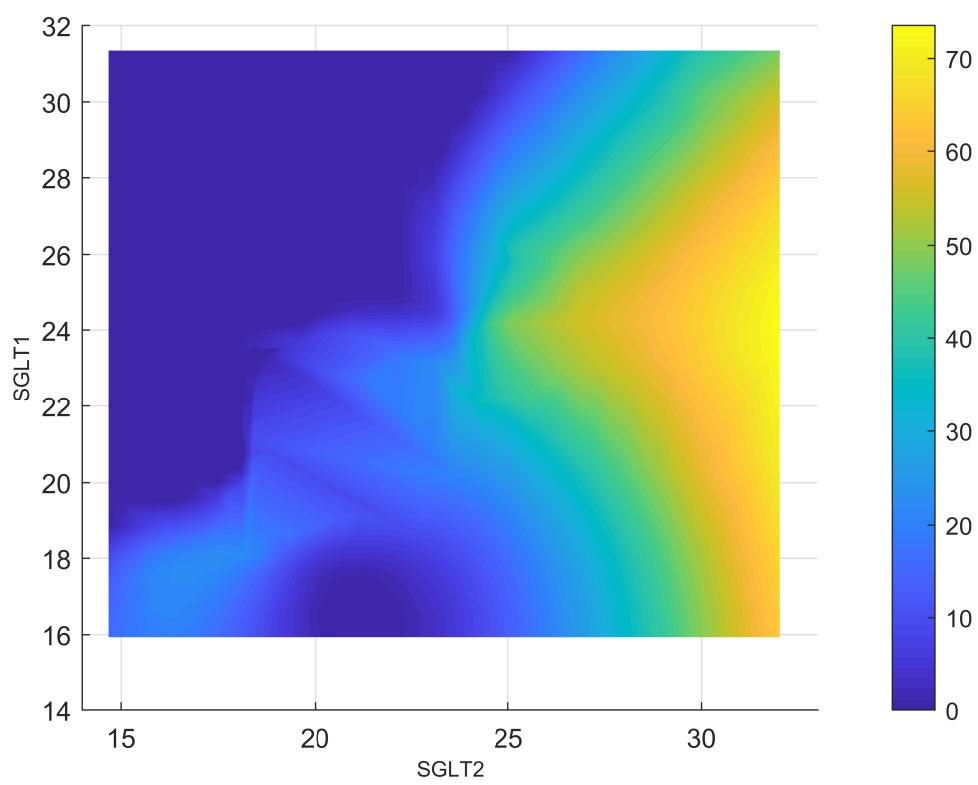

Supplement: Supplemental Information 1 [file peerj-11-15647-s001.zip › Raw Data/Raw data related to interpolation algorithm/3.Glycogen content/A high-risk warning indicator for glycogen content.pdf]

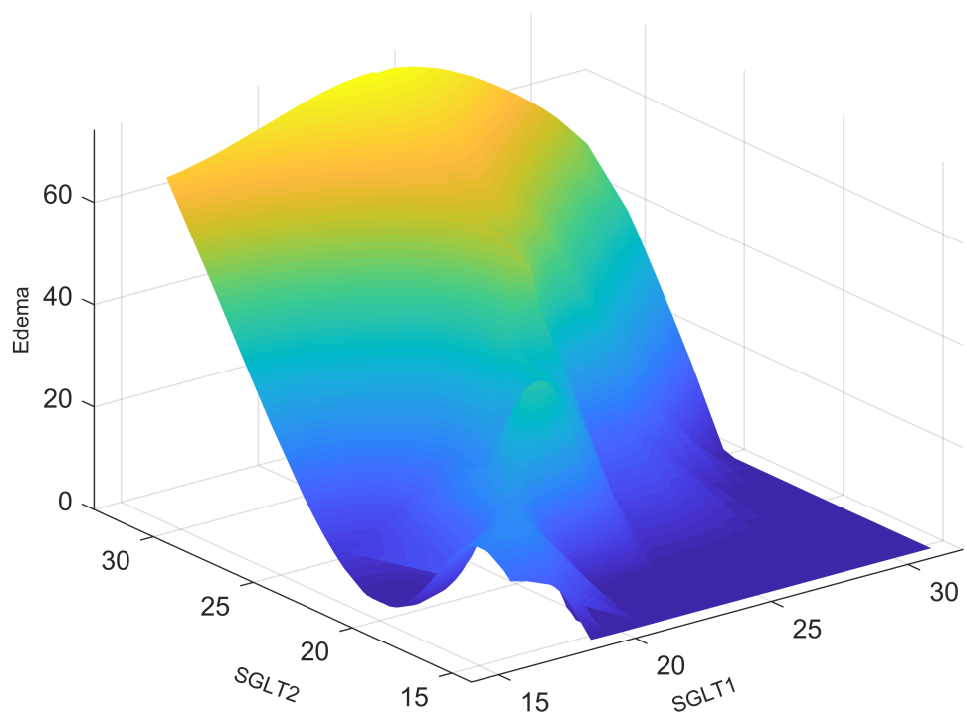

Supplement: Supplemental Information 1 [file peerj-11-15647-s001.zip › Raw Data/Raw data related to interpolation algorithm/3.Glycogen content/three-dimensional (3D) stereogram.pdf]

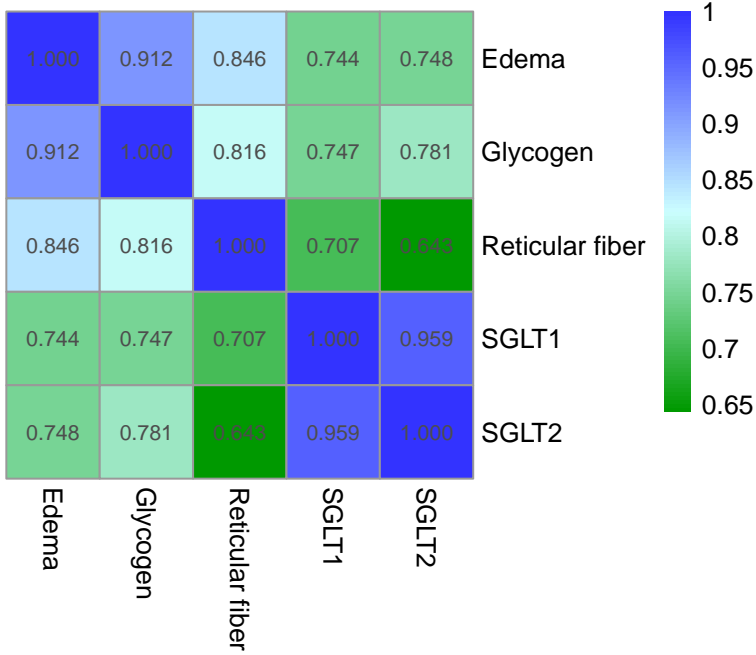

Supplement: Supplemental Information 1 [file peerj-11-15647-s001.zip › Raw Data/The strength of the correlation obtained by the code/all.cor_heatmap.pdf]

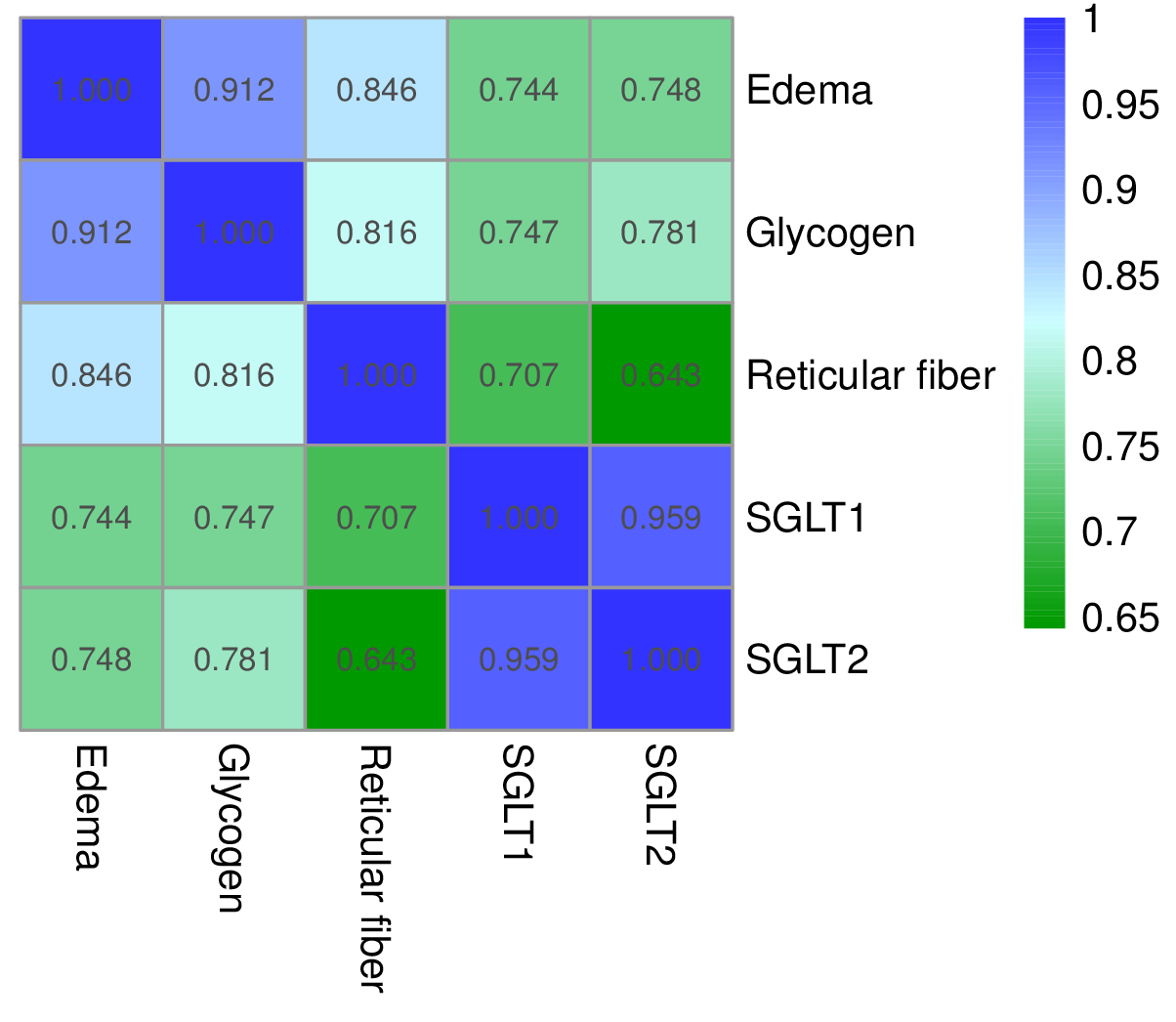

Supplement: Supplemental Information 1 [file peerj-11-15647-s001.zip › Raw Data/The strength of the correlation obtained by the code/all.cor_heatmap.png]
